# Responsiveness and minimum important change of the Pharmacotherapeutic Symptom Evaluation-20–Australian version: a tool for measuring changes in medicine-related symptoms over time

**DOI:** 10.1007/s11096-025-02045-4

**Published:** 2025-11-12

**Authors:** Abebe Basazn Mekuria, Andre Q. Andrade, Renly Lim, Debra Rowett, Mariann Hedström, Elizabeth E. Roughead

**Affiliations:** 1 Quality Use of Medicines and Pharmacy Research Centre, Clinical and Health Sciences, University of South Australia, Adelaide, 5001 Australia; 2 Centre for Translational Research, Institute for Research, Development and Innovation, IMU University, 57000 Kuala Lumpur, Malaysia; 3 Department of Public Health and Caring Sciences, Section of Caring Sciences, Uppsala University, BMC, Husargatan 3, 75122 Uppsala, Sweden

**Keywords:** Adverse drug events, Longitudinal monitoring, Minimum important change, Patient-reported outcomes, PHASE-20, Responsiveness, Survey

## Abstract

**Introduction:**

Monitoring patient-reported symptoms over time may support early detection of medicine-related harms. The Pharmacotherapeutic Symptom Evaluation-20–Australian version (PHASE-20–Australian version) is an 11-point rating scale designed for longitudinal monitoring of medicine-related symptoms; however, its responsiveness and minimum important change (MIC) have not been established.

**Aim:**

To evaluate the responsiveness and MIC of the PHASE-20–Australian version for monitoring changes in medicine-related symptoms over time.

**Method:**

A prospective cohort study was conducted among Australian adults (≥ 18 years) taking medications. Participants completed the PHASE-20–Australian version at baseline and follow-up and reported perceived symptom changes using the Global Rating Scale (GRS) at follow-up. Responsiveness was assessed by correlating score changes with the GRS and calculating the area under the receiver operating characteristic (ROC) curve (AUC). MIC was estimated using ROC anchor-based and 0.5 standard deviation (SD) distribution-based methods and then compared with the Smallest Detectable Change (SDC).

**Results:**

Among 102 participants, 52% were aged ≥ 60 years and 65.7% were female. Strong correlations were observed for overall score changes (*rho* = 0.815) with the GRS as well as for 84.2% of individual symptoms (*rho* = 0.701–0.897). The tool demonstrated good to strong discriminative ability (AUC = 0.739–0.975 for improvement; 0.764–0.977 for deterioration), with sensitivity and specificity ≥ 0.75 for 16 symptoms. The MIC values ranged from 0.5 to 1.5 using the ROC method and 0.9–1.8 using the 0.5 SD approach. The estimated MIC values exceeded the SDC for 84.2% of symptoms. Limited responsiveness (*rho* < 0.7, AUC < 0.7) and MIC values below the SDC were noted for forgetful, swollen legs/ankles and frequent urination/incontinent of urine.

**Conclusion:**

The PHASE-20–Australian version is responsive for most symptoms, with a clinically meaningful change of approximately 2.0 points on a 0–10 scale. The estimated MIC is applicable at the individual level, although caution is needed for symptoms with an MIC below the SDC.

**Supplementary Information:**

The online version contains supplementary material available at 10.1007/s11096-025-02045-4.

## Impact statements

- The findings of this study support the potential utility of the PHASE-20–Australian version for longitudinal monitoring of signs and symptoms of medicine-related harm in clinical practice or research.
- The MIC values established in this study provide useful benchmarks for informing study design and sample size estimation in clinical trials aimed at reducing medicine-related harm.
- The estimated MIC thresholds can support the interpretation of changes in medicine-related symptom burden and evaluation of interventions targeting medicine-related harm.
- The established MIC in this study could support clinical decision-making by distinguishing real from trivial symptom changes, promoting safer and more effective medicine use.
- The PHASE-20–Australian version could help pharmacists and clinicians track medicine-related symptoms over time, enhancing engagement and communication among healthcare providers in routine practice.

## Introduction

Adverse medicine events (AMEs) are prevalent in healthcare settings [1, 2], especially among patients taking multiple medications [3, 4]. Medicine-related harms are often unrecognized or underreported in routine clinical practice by both patients and healthcare practitioners [5, 6], and can lead to negative treatment outcomes [7, 8]. The under-reporting of AMEs in clinical practice underscores the need for more systematic approaches to detect AMEs [5, 9]. Strategies such as embedding monitoring of patient-reported outcomes into clinical practice may provide a systematic approach to capturing signals of medicine-related adverse events directly from patients, ensuring timely detection and intervention to prevent more serious medicine-related harm [10–12].

Patient reported outcomes, such as symptoms, can serve as early indicators of potential AMEs or medicine-related harm [13–15]. Studies have shown that between 53 and 72% of symptoms reported by patients were related to the medicines they were taking [16, 17]. Being alert to and longitudinally monitoring changes in such symptoms using patient-reported tool can support early detection and resolution of medicine-related harm and symptom burden [13, 18, 19]. Tools intended for longitudinal monitoring of patient-reported outcomes should be responsive to changes overtime and score changes should be interpretable through knowledge of the Minimum Important Change (MIC) [20–22]. Responsiveness refers to the ability of a measurement instrument to detect meaningful change over time [23], while the MIC represents the smallest change in score that patients perceive as important [22]. Establishing responsiveness and the MIC is essential to determine whether a tool can reliably detect meaningful changes over time and support its integration into clinical practice for longitudinal monitoring [22, 24, 25].

Patient-reported medicine-related symptom assessment tools, such as the Pharmacotherapeutic Symptom Evaluation-20 (PHASE-20), have been developed to identify potential medicine-related symptoms [26–28]. However, none have been designed or validated for the longitudinal monitoring of medicine-related symptoms [28]. The PHASE-20 was originally developed and validated to assess medicine-related symptoms among older adults in Sweden [27]. It is currently used as part of the Quality Indicators for Good Drug Therapy among elderly patients [29] and by pharmacists to identify medicine-related symptoms during medication reviews in community, primary care, and nursing home settings [30–32]. In a prior study, we adapted and pilot-tested the PHASE-20 by modifying its response format from a four-point scale to an 11-point numeric rating scale (PHASE-20–Australian version) for longitudinal monitoring of potential medicine-related symptoms [33]. The PHASE-20–Australian version consists of 19 predefined medicine-related symptoms and one open-ended question, with scores ranging from 0, indicating “no symptom,” to 10, representing the “worst possible symptoms” [33]. Evidence from our pilot study showed that the score changes in PHASE-20–Australian version demonstrated a good correlation (*rho* = 0.721) with the Edmonton Symptom Assessment System (a tool validated and widely used for longitudinal monitoring) [34], supporting its potential application for tracking symptoms over time [33]. The PHASE-20–Australian version can be used by healthcare professionals, including pharmacists, for the longitudinal monitoring of early signs and symptoms of medicine-related harm among patients who take medications regularly in routine practice. However, its ability to detect meaningful change over time and to identify the smallest changes perceived as clinically important by patients remains unknown.

## Aim

This study aimed to assess the responsiveness and MIC of the adapted PHASE-20–Australian version for monitoring changes in medicine-related symptoms over time.

## Method

### Study design

A prospective cohort study with a four-week follow-up was conducted in Australia to determine the responsiveness and MIC of the PHASE-20–Australian version for monitoring potential medicine-related symptoms over time.

### Participants inclusion and sample size

Adults aged 18 years or older, living in Australia and currently taking one or more medications (prescription or over the counter) regularly, were eligible to participate. Participants with incomplete responses and those who had discontinued all baseline medications and started entirely new medications at follow-up were excluded (i.e., participants were required to be taking at least one of the same medications throughout the follow-up period).

The sample size was determined using the rule of thumb outlined in established standards for studies on the measurement properties of PROMs, which recommend including approximately five to seven participants per item of the tested PROMs to obtain reliable results [22, 23, 25]. Based on this recommendation, since the tested tool comprises 20 items (19 symptom items and one open-ended question), the minimum sample size was estimated to be around 100 (5 × 20 items) and the maximum approximately 140 (7 × 20 items).

### Recruitment and data collection

Recruitment and data collection were conducted online using Qualtrics XM [35]. Survey access links and QR codes were posted and advertised on the University of South Australia’s ‘research volunteers’ webpage to recruit participants. The survey link was also distributed through social media platforms, including Facebook, LinkedIn, and X. Study flyers were distributed or posted in consumer service provider organizations to recruit potential participants, including clinic and pharmacy waiting areas; at the University of the Third Age (a non-profit organization providing education and social activities for older adults); at Probus clubs (community groups for retired or semi-retired adults that promote social engagement and lifelong learning); and during a session of the Successful Ageing seminar organized by the University of South Australia.

Baseline data were collected using a survey that included three sections: (i) socio-demographic characteristics (ii) medicine-related symptoms rating scale (PHASE-20–Australian version) and (iii) medication currently used. After four weeks, participants who completed the first-round survey were invited to complete the second-round survey again. At follow-up, participants were invited to complete the PHASE-20–Australian version again and also asked to rate how much their symptoms had changed over the four weeks since completing the first-round survey, using the Global Rating Scale (GRS) [22]. The GRS is a single-item, self-rated scale that captures participants’ perceived change in symptoms since baseline, using a seven-point response format: ‘much better’, ‘better’, ‘a little better’, ‘about the same’, ‘a little worse’, ‘worse’, or ‘much worse’ [22]. The GRS was selected because of its high face and content validity in capturing patient-perceived changes over time, its low respondent burden, its ability to reflect clinically meaningful changes from the patient’s perspective, and its assessment of the same construct as the instrument under study [36, 37]. We used the GRS as a comparator or external anchor for measuring change over time to determine the responsiveness and estimate the MIC of the adapted tool [36]. The GRS is widely recognized for its simplicity, sensitivity to subjective changes, and relevance to patient-reported outcomes [38]. While other approaches, such as objective clinical measures or repeated symptom scores, exist, these may not fully capture patient-perceived changes needed to estimate the smallest meaningful change from the patient’s perspective [24, 39].

Participants were also asked to indicate any changes in their medicine use since completing the first-round survey at follow up. Each participant’s follow-up survey was consistently administered four weeks after completing the baseline survey, ensuring uniform follow-up timing across all participants. The survey content, data collection procedures, and inclusion criteria remained unchanged throughout the entire study period. The four-week follow-up period was considered a reasonable timeframe to capture potential medicine-related symptoms, as previous research has shown that most medicine-related events are reported within four weeks of initiating a medication [40].The online survey was open from July 2024 to April 2025.

### Statistical analysis

Descriptive statistics were conducted to summarise sociodemographic characteristics, medication use and change scores on the PHASE-20–Australian version. All statistical analyses were performed using the R (version 4.2.2; R Foundation for Statistical Computing, Vienna, Austria).

### Determination of responsiveness

The responsiveness of the PHASE-20–Australian version was evaluated by assessing the correlation between the change in symptom scores and patient-perceived changes on the GRS. Due to non-normal data distribution, Spearman’s correlation coefficient was used [22, 41]. We examined correlations for both the overall PHASE-20 score and individual symptom items against their corresponding GRS ratings. A correlation ≥ 0.7 indicating acceptable responsiveness [41].

### Determination of MIC and discriminative ability

We estimated the MIC for each symptom in the PHASE-20–Australian version using both anchor-based and distribution-based methods [42, 43]. Anchor-based approaches determine the MIC by linking score changes to an external anchor [22, 44, 45], while distribution-based approaches estimate the MIC based on score variability using statistical methods [22, 46]. The MIC was estimated separately for improvement and deterioration, as the values may differ [45]. MIC was estimated for each symptom of the PHASE-20–Australian version. Symptoms that demonstrated at least a moderate correlation (r ≥ 0.30) with the selected anchor (GRS) were then included in the MIC estimation [24, 47].

A. *MIC estimation using anchor-based methods*
  We conducted a Receiver Operating Characteristic (ROC) analysis to determine the MIC for both improvement and deterioration [22, 42]. For the ROC analysis, participants were divided into three groups based on their responses to the GRS: those who reported feeling much better, better, or a little better were categorized as the ‘Improved’ group; those who reported feeling about the same were categorized as the ‘Unchanged’ group; and those who reported feeling a little worse, worse, or much worse were classified as the ‘Deteriorated’ group. ROC curves were then plotted with the true positive rate (sensitivity) on the y-axis and the false positive rate (1–specificity) on the x-axis [22], separately for improvement and deterioration. The optimal cut-off point (MIC) for improvement and deterioration of each potential medicine-related symptom was selected based on the highest Youden’s J index [48] and then confirmed using the top-left approach (the point of the shortest distance to the top-left corner of the ROC curve) [22, 49]. The AUC was calculated to evaluate the tool’s discriminative ability [22], with values ≥ 0.70 indicating good performance and further supporting its responsiveness [50, 51].

B. *MIC estimation using distribution-based approach*
  We calculated the 0.5 standard deviation (SD) to estimate the MIC to support the anchor-based MIC values [52].

### Comparison of estimated MIC and smallest detectable change (SDC)

The estimated MIC values were compared with the corresponding SDC to determine whether the PHASE-20 Australian version is reliable enough to detect changes beyond measurement error, thereby supporting its application at the patient level [22, 53, 54]. The SDC, representing the smallest real change beyond measurement error, was calculated using the formula SDC = 1.96 × √ 2 × SEM, where 1.96 reflects the 95% confidence level, √ 2 accounts for variance across two time points (baseline and follow-up), and SEM (Standard Error of Measurement) was derived as SEM = SD × √ (1 − r), with SD as the standard deviation of change scores and r (test–retest reliability) estimated using the intraclass correlation coefficient (ICC) from participants who reported ‘about the same’ on the GRS [25].

## Ethics approval

Ethics approval was obtained from the Human Research Ethics Committee of the University of South Australia (Application No. 205895) on June 25, 2024.

### Results

#### Sociodemographic characteristics

Out of the 214 respondents who completed the baseline assessment, 137 agreed to participate in the follow-up study. A total of 106 participants submitted responses to the follow-up survey, of which 4 were excluded because they did not complete the tested tool (PHASE-20–Australian version) section of the survey. Of the 102 participants, 52% were aged 60 years or older, and 66% were female. Participants used an average of 6.5 medications, with an SD of 3.2. Using the PHASE-20–Australian version, participants reported a median (IQR) of 10.0 (9.0–13.0) potential medicine-related symptoms (Table 1).

**Table 1:** The sociodemographic characteristics of participants who used one or more medicines over four weeks (N = 102)

Characteristics	Frequency
Age, n (%)	
< 60 years (22–59 years)	49 (48.0)
≥ 60 years (60–86 years)	53 (52.0)
Gender, n (%)	
Male	35 (34.3)
Female	67 (65.7)
Type of medicines used by participants*, N = 102	
Prescription medicines, n (%)	99 (97.1)
Over the counter medicines, n (%)	38 (37.3)
Number of medicines	
< 5	46 (45.1)
≥ 5	56 (54.9)
Number of medicines used per participants (Mean ± SD), (minimum, maximum)	6.5 ± 3.2, (1–14)
The most frequently used medicine classes (N = 102)	
HMG CoA reductase inhibitors	34 (33.3)
Proton pump inhibitors (PPIs)	27 (26.5)
Selective serotonin reuptake inhibitors (SSRIs)	21 (20.6)
Analgesic (non-opioid analgesic)	19 (18.6)
Angiotensin II receptor blockers (ARBs)	18 (17.7)
Beta-blocking agents	18 (17.7)
Biguanides	16 (15.7)
Platelet aggregation inhibitors	14 (13.7)
ACE inhibitors	12 (11.8)
SGLT-2 inhibitors	11 (10.9)
Serotonin-norepinephrine reuptake inhibitors (SNRIs)	10 (9.8)
The most frequently used individual medicine (N = 102)	
Metformin	19 (18.6)
Rosuvastatin	18 (17.7)
Atorvastatin	16 (15.7)
Perindopril	13 (12.8)
Empagliflozin	12 (11.8)
Aspirin	8 (7.8)
Metoprolol	8 (7.8)
Thyroxine	8 (7.8)
Amitriptyline	7 (6.9)
Amlodipine	7 (6.9)
Escitalopram	7 (6.9)
Changes in potential medicine-related symptoms over four weeks reported using PHASE-20–Australian version, n (%)	
Increased severity scores	48 (47.1)
Decreased severity scores	42 (41.2)
Unchanged severity scores	12 (11.8)
Total reported potential medicine related symptoms, median (IQR), (minimum, maximum)	10.0 (9.0, 13.0), (1, 19)

^*^Participants may be classified into more than one category, IQR, Interquartile range

#### Symptom changes over a four-week period

Table 2 presents the median change in symptom scores stratified by patient-reported global ratings of change (improved, no change, and deteriorated) for each symptom. All symptoms rated as ‘improved’ on the GRS had positive median change scores on the PHASE-20–Australian version, whereas those rated as ‘deteriorated’ had negative median change scores (Table 2).

**Table 2:** Changes in PHASE-20–Australian version symptom scores from baseline to 4 weeks, stratified by GRS categories (N = 102) (Table 2)

Potential medicine related symptoms	PHASE-20–Australian version change scores stratified by GRS					
	Improved (n = 31)		No Change (n = 30)		Deteriorated (n = 41)	
	n	Median changes (IQR)	n	Median changes (IQR)	n	Median changes (IQR)
Dizzy/unsteady	14	2.0 (1.0, 2.3)	14	0.0 (− 1.0, 1.0)	37	− 2.0 (− 4.0, 1.5)
Tired/exhausted	33	2.0 (2.0, 4.0)	26	0.0 (0.0, 0.3)	41	− 2.0 (− 4.0, 2.0)
Poor sleep patters	29	2.0 (1.0, 4.0)	34	0.0 (− 0.3, 1.0)	30	− 3.0 (− 4.0, − 2.0)
Abdominal pain/chest pain	17	2.0 (0.5, 5.0)	22	0.5 (− 1.0, 2.0)	22	− 2.0 (− 3.0, − 1.0)
Headache	23	3.0 (1.0, 5.0)	22	0.0 (− 1.0, 1.0)	20	− 2.0 (− 3.8, − 1.0)
Low mood	26	3.0 (2.0, 4.5)	24	0.0 (− 1.0, 1.0)	28	− 2.0 (− 3.0, − 2.0)
Worried/anxious	20	2.0 (2.0, 5.8)	30	1.0 (0.0, 1.3)	32	− 3.0 (− 4.8, − 1.0)
Irritable	19	3.0 (2.0, 4.0)	26	0.0 (− 1.0, 1.0)	31	− 2.0 (− 4.0, − 2.0)
Forgetful	15	0.0 (− 1.5, 2.5)	30	0.0 (− 1.0, 1.0)	18	− 2.0 (− 2.5, − 0.3)
Poor appetite	14	3.0 (2.0, 3.5)	16	− 1.0 (− 1.0, 0.0)	24	− 3.0 (− 4.0, − 1.0)
Dry mouth	21	3.0 (2.0, 6.0)	24	0.0 (− 1.0, 1.0)	31	− 2.0 (− 5.0, − 2.0)
Nausea/vomiting	17	3.0 (2.0, 6.0)	16	0.0 (− 1.0, 1.0)	18	− 2.0 (− 4.0, 1.8)
Diarrhea	11	3.0 (2.0, 6.0)	10	0.5 (0.0, 1.3)	9	− 2.0 (− 4.0, − 1.0)
Constipation	17	3.0 (1.5, 5.0)	16	0.0 (0.0, 1.0)	18	− 3.0 (− 4.3, − 1.8)
Palpitation (rapid/irregular heartbeat)	18	4.0 (1.5, 4.5)	16	1.0 (− 1.5, 2.0)	10	− 2.5 (− 4.0, 1.8)
Swollen legs/ankles	12	1.0 (− 0.3, 2.3)	10	1.0 (− 1.0, 1.0)	10	− 1.0 (2.3, 0.3)
Shortness of breath	17	2.0 (1.0, 3.0)	12	0.0 (− 1.0, 1.0)	13	− 2.5 (4.0, − 1.0)
Frequent urination/incontinent of urine	19	3.0 (− 0.5, 5.0)	25	0.0 (− 2.0, 1.0)	15	− 2.0 (− 5.0, 0.0)
Itching/rash	24	2.0 (1.0, 3.8)	15	0.0 (− 1.0, 0.0)	23	− 3.0 (− 4.8, − 2.0)

#### Determination of responsiveness of the PHASE-20–Australian version

**Fig. 1:**
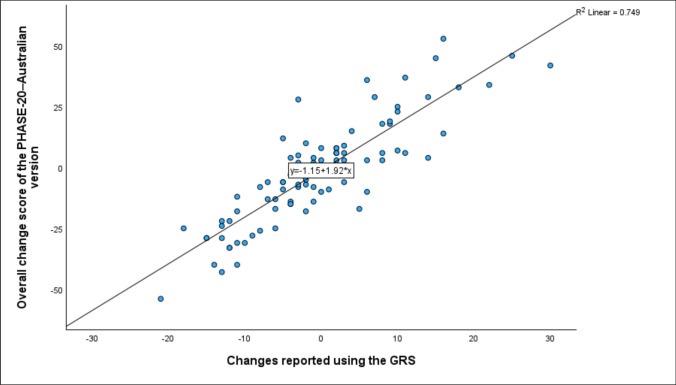
The correlation between the overall score changes in the PHASE-20–Australian version (from baseline to the four-week follow-up) and GRS scores (N = 102)
B. *Responsiveness of the tested tool for individual potential medicine-related symptoms*
  All potential medicine-related symptoms listed in the PHASE-20–Australian version demonstrated a correlation above 0.3 with perceived changes reported using the GRS. The tool showed acceptable to strong responsiveness for the majority (84.2%) of potential medicine-related symptoms (*rho* = 0.701–0.897), except for symptoms: forgetful, frequent urination/incontinent of urine, and swollen legs/ankles, which had correlations below 0.50 (Table 3).

A. *Correlation between overall score changes in tested tool and GRS*
  A strong positive correlation was found between the overall change score of the PHASE-20–Australian version and perceived symptom changes reported using the GRS (*rho* = 0.815, *p* < 0.001), indicating good responsiveness of the tool (Fig. 1).

**Table 3:** Responsiveness of the PHASE-20–Australian version: correlation between changes score in individual symptom and GRS scores (N = 102)

Symptoms for which a change was reported using PHASE-20–Australian version	Participants reported changes in symptom scores on the PHASE-20–Australian version and the GRS, n	Correlation between score changes in the PHASE-20–Australian version and the GRS, Spearman’s *rho*
Dizzy/unsteady	65	0.806
Tired/exhausted	100	0.897
Poor sleep pattern	93	0.704
Abdominal pain/chest pain	61	0.713
Headache	65	0.710
Low mood	78	0.831
Worried/anxious	82	0.786
Irritable	74	0.847
Forgetful	63	0.353
Poor appetite	52	0.755
Dry mouth	75	0.868
Nausea/vomiting	51	0.876
Diarrhea	30	0.852
Constipation	51	0.760
Palpitations (rapid/irregular heartbeat)	41	0.837
Swollen legs/ankles	32	0.345
Shortness of breath	43	0.701
Frequent urination/incontinent of urine	59	0.455
Itching/rash	67	0.878

#### MIC and discriminative ability of the PHASE-20–Australian version

A. *Estimation of MIC using ROC method*
  Table 4 presents the MIC values and discriminative ability of the PHASE-20–Australian version for identifying both improvement and deterioration in medicine-related symptoms, based on the ROC analysis approach. The majority (84.2%) of symptoms showed acceptable to excellent discriminative ability (AUC ≥ 0.7), ranging from 0.739 to 0.975 for improvement and 0.764–0.977 for deterioration. Weak discriminative ability (AUC < 0.7) was observed for symptoms: forgetful, swollen legs/ankles, and frequent urination/incontinent of urine (Table 4). For improvement, 15 out of 19 potential medicine-related symptoms had a MIC cut-off of 1.5 points (sensitivity: 0.333–0.905; specificity: 0.503–0.975), while four symptoms had a cut-off of 0.5 points (sensitivity: 0.678–0.929; specificity: 0.698–0.944). For deterioration, 13 symptoms had a 1.5-point cut-off (sensitivity: 0.600–0.929; specificity: 0.682–0.933), and seven had a 0.5 points cut-off (sensitivity: 0.800–0.973; specificity: 0.625–0.933) (Table 4)

**Table 4:** MIC and discriminative ability of the PHASE-20–Australian version using ROC method for improvement and deterioration of potential medicine related symptoms (N = 102)

Reported symptoms	MIC for improvement				MIC for deterioration			
	Sensitivity	Specificity	AUC	Cut-off	Sensitivity	Specificity	AUC	Cut-off
Dizzy/unsteady	0.929	0.743	0.865	0.50*	0.973	0.733	0.925	1.50
Tired/exhausted	0.879	0.962	0.975	1.50	0.927	0.923	0.934	0.50
Poor sleep pattern	0.754	0.941	0.898	1.50	0.830	0.765	0.966	0.50
Abdominal pain/chest pain	0.771	0.955	0.739	1.50*	0.955	0.682	0.843	1.50
Headache	0.752	0.909	0.770	1.50	0.700	0.909	0.836	1.50
Low mood	0.885	0.830	0.901	1.50	0.821	0.875	0.914	1.50
Worried/anxious	0.800	0.767	0.803	1.50	0.906	0.800	0.902	1.50
Irritable	0.824	0.885	0.889	1.50	0.871	0.923	0.942	1.50
Forgetful	0.444	0.795	0.503	1.50	0.667	0.846	0.637	1.50*
Poor appetite	0.923	0.933	0.921	0.50	0.767	8.400	0.839	1.50
Dry mouth	0.905	0.870	0.958	1.50	0.774	0.826	0.852	1.50
Nausea/vomiting	0.833	0.787	0.934	1.50	0.778	0.875	0.866	1.50
Diarrhea	0.818	0.800	0.850	1.50	0.889	0.900	0.950	0.50
Constipation	0.765	0.937	0.801	1.50	0.889	0.811	0.924	0.50
Palpitations (rapid/irregular heartbeat)	0.692	0.889	0.880	1.50	0.800	0.889	0.894	1.50
Swollen legs/ankles	0.333	0.423	0.594	1.50	0.800	0.625	0.673	0.50
Shortness of breath	0.757	0.904	0.802	1.50*	0.714	0.864	0.807	1.50
Frequent urination/incontinent of urine	0.578	0.686	0.598	0.50	0.600	0.714	0.690	1.50*
Itching/rash	0.875	0.780	0.944	0.50	0.929	0.933	0.977	1.50

AUC, Area under the receiver-operating characteristic curve; MI, Minimum important change

^*^The cut-off value was selected based on the smallest top-left value, as multiple cut-off points had equally high Youden’s J index values
B. *MIC estimation using distribution-based approach*
  Table 5 presents the MIC values determined using distribution-based approaches. The MIC values varied across symptoms, ranging from 0.9 to 1.8 points on the 0–10 scale, with most symptoms (14 out of 19) falling between 1.3 and 1.5 points (Table 5).

**Table 5:** MIC estimated using distribution-based approaches for potential medicine-related symptoms using the PHASE-20–Australian version (N = 102)

Potential medicine-related symptoms	Estimated MIC using distribution-based approaches (0.5 SD)
Dizzy/unsteady	1.0
Tired/exhausted	1.4
Poor sleep pattern	1.4
Abdominal pain/chest pain	1.3
Headache	1.5
Low mood	1.5
Worried/anxious	1.4
Irritable	1.4
Forgetful	1.3
Poor appetite	1.4
Dry mouth	1.8
Nausea/vomiting	1.4
Diarrhea	1.7
Constipation	1.7
Palpitation (rapid or irregular heartbeat)	1.6
Swollen legs/ankles	0.9
Shortness of breath	1.5
Frequent urination/incontinent of urine	1.5
Itching/rash	1.3

SD, Standard deviation

C. *Comparison of estimated MIC with SDC*
  For the majority of potential medicine-related symptoms (16 out of 19) listed in the PHASE-20–Australian version, the estimated MIC values were greater than their corresponding SDC. Symptoms including forgetful, swollen legs/ankles and frequent urination/incontinent of urine showed MIC values smaller than the SDC (a detailed comparison is presented Supplementary 1).

## Discussion

This study evaluated the responsiveness and MIC of the PHASE-20–Australian version and its applicability at the individual patient level for detecting changes over time in potential medicine-related symptoms. The PHASE-20–Australian version demonstrated acceptable to strong responsiveness for the majority of potential medicine-related symptoms, with a change of approximately 1.0–2.0 points on the 0–10 scale generally required to reflect a clinically meaningful change. The estimated MIC values for majority of symptoms in the PHASE-20–Australian version was greater than their corresponding SDC values, supporting the interpretability of changes at the individual patient level. The findings of this study support the potential utility of the PHASE-20–Australian version for longitudinal monitoring in clinical practice or research.

In our study, the change in scores of the PHASE-20–Australian version showed the expected patterns across GRS categories, with improved symptoms exhibiting positive median change scores and deteriorated symptoms showing negative median change scores. The score changes on the PHASE-20–Australian version showed acceptable to strong correlation with GRS scores (*rho* = 0.704–0.897) for the majority of symptoms, indicating that the tool demonstrated acceptable to high responsiveness. This finding was supported by AUC values, where the majority of symptoms showed acceptable to good discriminative ability for both improvement (AUC = 0.739–0.975) and deterioration (0.764–0.977). These findings indicate that the PHASE-20–Australian version can detect meaningful changes over time in the majority of symptoms [22, 24, 25]. Evidence from previous studies [24, 25] and the guideline by de Vet et al. [22] on measuring PROMs indicates that the AUC or the correlation should be greater than 0.7 to conclude that the tested PROM has good responsiveness and supports its use for longitudinal monitoring [22, 24]. In our study, however, some symptoms such as forgetful, swollen legs/ankles and frequent urination/incontinent of urine, showed weak correlations with patient-perceived changes reported using the GRS (*rh*o = 0.345–0.455) and low AUC values (AUC < 0.7). This suggests that the PHASE-20–Australian version may be less sensitive in detecting changes over time for these specific symptoms among the general population. The low responsiveness may be due to the unpredictable nature of symptoms or side effects, which can spontaneously appear or resolve regardless of the medication being used [55, 56]. A prior study on the importance and methodological challenges of patient-reported questionnaires for side effects highlighted the difficulty of capturing fluctuating symptom or side effects patterns within fixed follow-up periods [56].

In our study, MIC was estimated for each symptom listed in the PHASE-20–Australian version, as all symptoms demonstrated correlations greater than 0.30 with the GRS. Consistent with methodological guidelines on evaluation of measurement properties of PROMs, a correlation exceeding 0.30 is generally considered to indicate a meaningful change perceived by patients rather than random variation [22, 24, 57]. As recommended in the guideline by De Vet et al. [22], we estimated the MIC for the PHASE-20–Australian version using both anchor-based (ROC method) and distribution-based approaches to enhance the reliability of the estimated MIC. The ROC analysis in our study identified MIC cut-off points ranging from 0.5 to 1.5 points on the PHASE-20–Australian version for both improvement and deterioration across symptoms, suggesting that a change of approximately 1.0–2.0 points on the 0–10 scale is generally required to reflect a clinically meaningful change. The ROC-based MIC is particularly useful for interpreting individual-level changes, as it provides information on sensitivity, specificity, and discriminative ability (AUC) [22, 45]. However, this method does not account for variability in scores within the sample or changes in direction (improvement or deterioration) [22]. To address this limitation, we also estimated MIC values using the distribution-based method, which ranged from 0.9 to 1.8 points across potential medicine-related symptoms, consistent with the MIC estimates obtained from the anchor-based method. This consistent MIC results from both anchor-based and distribution-based methods support the reliability of the estimated MIC values. Studies have indicated that using both methods leverages their respective strengths, enhancing the reliability of the estimated MIC values as well as their interpretability and applicability in clinical and research contexts [22, 45, 58].

For an MIC to be applicable at the individual level, as recommended in published guidelines and studies, it should exceed the corresponding SDC and demonstrate sensitivity and specificity of at least 0.75, indicating a true change beyond measurement error and reduced misclassification of patients [22, 53, 54]. In our study, the estimated MIC for most symptoms exceeded the SDC and met the recommended thresholds, suggesting that the PHASE-20–Australian version can reliably detect true changes over time at the individual patient level. However, for symptoms such as forgetful, swollen legs/ankles and frequent urination/incontinent of urine, the estimated MIC values were below the SDCs and exhibited low sensitivity or specificity (< 0.50), suggesting a high probability of misclassification and limited ability to detect meaningful changes beyond measurement error for these symptoms in the study population based on single measurements [22]. The low MIC values for these symptoms may be attributed to the small subgroup sample or variability within the study population, as the PHASE-20 was originally designed for older adults in nursing homes [27], where perceived changes in these symptoms may differ from those in the general population.

The MIC values estimated in this study can be valuable for informing study design and sample size estimation in clinical trials focused on reduction of medicine related harm in practice. The identified MIC thresholds can also be applied at the individual patient level to facilitate the interpretation of changes in medicine-related symptom burden and to evaluate interventions targeting medicine-related harm using medicine-related adverse events as primary outcomes. Evaluating treatment efficacy based solely on statistical significance may not accurately reflect patient benefit [59]. In contrast, the concept of the MIC provides a threshold for the smallest change in an outcome that patients perceive as benefit [60].

### Limitation of the study

To the best of our knowledge, no previous studies have reported the MIC and its application at the individual patient level for a tool designed to monitor medicine-related symptoms over time. However, the following limitations should be considered when interpreting the findings of this study. We did not perform a formal causality assessment reported adverse events due to limited data and, therefore, cannot rule out the possibility that changes in reported symptoms may reflect fluctuations in overall illness burden rather than being related to medication use. A potential limitation is the use of the single-item GRS, which may be prone to recall bias and reflect recent rather than baseline health status [22, 61, 62]. Another limitation of this study is the participant dropout observed during the follow-up period (22.6% dropout rate), which may be attributed to the online self-administered survey format, participants’ health conditions, fear of sharing information online, loss of interest, or competing personal commitments. Efforts were made to encourage continued participation through reminder emails and by extending the online participants recruitment period to achieve the minimum required sample size. The final sample size (n = 106) remained within the recommended range for evaluating responsiveness and estimating the MIC. However, the reduced sample size may have limited the statistical power of the subgroup analyses for determining the MIC of individual symptoms, which may have affected the stability of the estimated MIC and SDC for individual symptoms. Despite these limitations, this study provides initial evidence supporting the responsiveness and MIC of the PHASE-20–Australian version, as well as its application in detecting changes in potential medicine-related symptoms over time at the individual level among patients who use medications.

## Conclusion

The PHASE-20–Australian version is responsive for the majority of potential medicine-related symptoms, except for the symptoms: forgetful, Frequent urination/incontinent of urine, and swollen legs/ankles. Given the variable MIC ranged up to 1.8, a change of approximately 2.0 points on the 0–10 scale could be considered reflective of clinically meaningful change in practice. The estimated MIC values were applicable at the individual level for the majority of symptoms but require caution for symptoms with an estimated MIC less than the SDC with sensitivity or specificity below 0.75, and an AUC less than 0.7. Further research with larger and more diverse population is required to enhance generalizability and refine symptom-specific MIC thresholds.

## Supplementary Information

Below is the link to the electronic supplementary material.

Supplementary file1 (DOCX 24 KB)

## Data Availability

Data are available upon reasonable request from the corresponding author.
