# Supplementary material for: Responsiveness and minimum important change of the Pharmacotherapeutic Symptom Evaluation-20–Australian version: a tool for measuring changes in medicine-related symptoms over time
Source: Int J Clin Pharm. 2025 Nov 12;48(2):644–55. doi: 10.1007/s11096-025-02045-4 (PMC12992458; doi:10.1007/s11096-025-02045-4)
Supplement: Supplementary file 1 — Supplementary file1 (DOCX 24 KB) [file 11096_2025_2045_MOESM1_ESM.docx]

**Comparison of estimated MIC with SDC**

For the majority of potential medicine-related symptoms (16 out of 19) listed in the PHASE-20 Australian version, the MIC values were greater than their corresponding SDC. However, a few symptoms including forgetful, frequent urination or incontinence, and swollen legs or ankles showed MIC values smaller than the SDC (Supplementary 1).

Supplementary 1: Estimated MIC potential medicine-related symptoms based on anchor- and distribution-based approaches, and comparison with SDC values.

| **Symptoms reported using PHASE-20–Australian version** | **Estimated MIC** | | | **Smallest Detectable Changes (SDC)** | **Potential application of the estimated MIC at individual patients’ level** |
| --- | --- | --- | --- | --- | --- |
|  | **MIC estimated using ROC anchor method** | | **MIC estimate using distribution method** |  |  |
|  | **Estimated MIC for improvement** | **Estimated MIC for deterioration** |  |  |  |
| Dizzy/unsteady | 0.50* | 1.50 | 1.0 | 0.5 | Yes |
| Tired/exhausted | 1.50 | 0.50 | 1.4 | 0.4 | Yes |
| Poor sleep pattern | 1.50 | 0.50 | 1.4 | 0.5 | Yes |
| Abdominal pain/chest pain | 1.50* | 1.50 | 1.3 | 1.2 | Yes |
| Headache | 1.50 | 1.50 | 1.5 | 1.3 | Yes |
| Low mood | 1.50 | 1.50 | 1.5 | 0.9 | Yes |
| Worried/anxious | 1.50 | 1.50 | 1.4 | 1.0 | Yes |
| Irritable | 1.50 | 1.50 | 1.4 | 1.1 | Yes |
| Forgetful | 1.50 | 1.50* | 1.3 | 3.0 | With caution |
| Poor appetite | 0.50 | 1.50 | 1.4 | 0.4 | Yes |
| Dry mouth | 1.50 | 1.50 | 1.8 | 1.4 | Yes |
| Nausea/vomiting | 1.50 | 1.50 | 1.4 | 1.3 | Yes |
| Diarrhea | 1.50 | 0.50 | 1.7 | 0.5 | Yes |
| Constipation | 1.50 | 0.50 | 1.7 | 0.5 | Yes |
| Palpitations (rapid/irregular heartbeat) | 1.50 | 1.50 | 1.6 | 1.4 | Yes |
| Swollen legs/ankles | 1.50 | 0.50 | 0.9 | 1.7 | With caution |
| Shortness of breath | 1.50* | 1.50 | 1.5 | 1.4 | Yes |
| Frequent urination/incontinent of urine | 0.50 | 1.50* | 1.5 | 2.9 | With caution |
| Itching/rash | 0.50 | 1.50 | 1.3 | 0.5 | Yes |

* The cut-off value was selected based on the smallest top-left value, as multiple cut-off points had equally high Youden’s J index values; Abbreviations: AUC=area under the receiver-operating characteristic curve; MIC=Minimum Important Change; SD = standard deviation.
